# In Vitro Effect of Anodization of Titanium Abutments on Color Parameters and Color Difference of Lithium Disilicate All‐Ceramic Crowns

**DOI:** 10.1002/cre2.70002

**Published:** 2024-09-19

**Authors:** Sotude Khorshidi, Arash Zarbakhsh, Shirin Lawaf, Shaghayegh Golalipour, Maryam Sayyari, Alireza Mahmoudi Nahavandi

**Affiliations:** ^1^ Department of Prosthodontics, Dental Branch Islamic Azad University Tehran Iran; ^2^ Department of Fixed Prosthodontics, Dental Branch Azad University Tehran Iran; ^3^ Department of Dental Materials Tehran University of Medical Sciences Tehran Iran; ^4^ Institute for Color Science and Technology Tehran Iran

**Keywords:** ceramics, color, dental implants, dental prosthesis, titanium

## Abstract

**Objective:**

This study assessed the effect of the anodization of titanium abutments on the color parameters and color difference of lithium disilicate (LDS) all‐ceramic crowns.

**Materials and Methods:**

In this study, 19 straight abutments were divided into two groups: anodized (*n* = 9) and non‐anodized control (*n* = 9), with one hybrid zirconia abutment as a reference. Anodization was achieved by applying 63 V energy using seven 9 V flat batteries in series, with an electrolyte solution comprising 1 g trisodium phosphate in 250 mL distilled water for 5 s, resulting in a gold‐yellow color. Abutments were then scanned, and full‐contour monolithic IPS e.max maxillary central incisor crowns were fabricated with 2 mm thickness and glazed. Reflectance was measured using a spectroradiometer, and color coordinates (L*, a*, b*, h*, and C*) were calculated using CS‐10W software. Color differences of the crowns in both groups were quantified using the CIEDE2000 (Δ*E*_00_) color difference formula and analyzed by *t*‐test (*α* = 0.05) compared to the standard sample.

**Results:**

The L*, a*, b*, and c* parameters in anodized abutments were significantly higher than those in non‐anodized abutments, while the h* parameter in anodized abutments was significantly lower than that in non‐anodized abutments (*p* < 0.001 for all). There was a significant difference in Δ*E*_00_ of the two groups (*p* = 0.043).

**Conclusion:**

Anodization of titanium abutments improved the color parameters of LDS all‐ceramic crowns and significantly decreased their Δ*E* compared with non‐anodized abutments.

## Clinical Relevance

The research findings indicate that the anodization of titanium abutments significantly impacts color parameters, leading to lighter, redder, and more color‐saturated samples compared to non‐anodized ones. This suggests that anodization can optimize color matching in dental restorations, particularly in esthetic dentistry where precise color reproduction is crucial for natural‐looking results. Implementing anodization techniques may enhance the overall esthetic outcomes of dental prosthetics, providing clinicians with a valuable tool to improve patient satisfaction and confidence in their smiles.

## 1 Introduction

Titanium abutments are the gold standard for replacing lost teeth with dental implants due to their optimal mechanical and biological properties (McGlumphy, Robinson, and Mendel 1992; Dede et al. 2016). However, their gray metallic color may be visible through the mucosa and under prosthetic crowns, potentially compromising esthetics, even when using all‐ceramic restorations (Jung et al. 2007; Park et al. 2007). To address this issue, one approach is to increase the thickness and opacity of the ceramic or consider changing the type of abutment used. Different materials may be used to fabricate abutments, such as gold, titanium nitride, alumina, zirconia, and composite resin‐coated titanium to improve esthetics. However, these alternatives have limited clinical applications due to several drawbacks (Linkevicius and Vaitelis 2015; Blatz et al. 2009; Kim et al. 2016; Cosgarea et al. 2015; Zembic et al. 2009; Sicilia et al. 2015; Jirajariyavej, Wanapirom, and Anunmana 2018; Dede et al. 2013).

Anodization has been proposed to enhance the esthetic appeal of titanium abutments. It is an electrochemical process that thickens and alters the appearance of the natural oxide layer on the titanium surface. This layer forms when titanium is exposed to an electrolyte solution with varying voltages (ranging from 5 to 90 V) (Wang et al. 2019). This layer can exhibit various colors because light interacts with the titanium dioxide layer. Significantly, this process does not alter the chemical composition of titanium; it solely increases the thickness of the oxide layer. The resulting color is stable and offers long‐term durability, mainly when covered by a prosthetic crown or soft tissue (Sharma 1992). Optimal biocompatibility, increased wear resistance and corrosion resistance, ￼ improvement of titanium surface color stability, ￼ enhancement of surface roughness, and subsequently increased retention of cement are among the advantages of anodization (Wadhwani et al. 2018). Studies have shown that anodized pink and gold abutments improve the esthetics of peri‐implant soft tissues compared to non‐anodized titanium abutments (Wang et al. 2019, 2021). Additionally, coloring the abutment neck, especially in light pink, can notably enhance peri‐implant gingival esthetics (Ishikawa‐Nagai et al. 2007). It has also been reported that utilizing a gold abutment with a zirconia coping represents the optimal choice for prosthetic crowns in the esthetic zone (Peng et al. 2017).

Most relevant available studies have focused on evaluating the effects of anodization of abutments on gingival esthetics (Wang et al. 2019, 2021; Ishikawa‐Nagai et al. 2007), with limited research on the impact of anodized abutments on color parameters of all‐ceramic crowns. Furthermore, these studies have typically utilized disc‐shaped specimens (Farrag, Bakry, and Aly 2022a, 2022b).

Given that natural teeth exhibit curvature, which can impact light absorption, transmission values, and consequently, color parameters (Hung 1990), this study aimed to investigate the effect of anodization of titanium abutments on the color parameters (L*, a*, b*, h*, and C*), and the color difference (Δ*E*) of LDS all‐ceramic crowns using teeth‐shaped specimens. The initial hypothesis of the study is that there is a significant difference in the ability of various titanium abutments, anodized or non‐anodized, and the standard abutment to be masked in color by LDS all‐ceramic crowns.

## 2 Materials and Methods

The sample size was calculated to be a minimum of nine specimens in each group, according to a study by Wang et al. (2021), assuming *α* = 0.05, *β* = 0.2, and a mean standard deviation of 3.5 to detect a 5‐unit difference between the two groups using PASS 11.0 software.

A total of 19 dental implants measuring 10 × 4.5 mm (Tixos MC, Leader Italia, Italy) and 19 straight abutments (Tixos MC, Leader Italia) with 5 mm diameter, 6 mm height, and 2 mm gingival height with Morse Taper connection were used in this study. The straight abutments were randomly assigned to two groups (*n* = 9) of anodization and control (no anodization), using the Rand feature of Excel software. Also, one zirconia hybrid abutment served as the standard group.

To simulate the clinical oral environment, dental implants were mounted in precise auto‐polymerizing acrylic resin molds (Meliodent; HeraeusKulzer GmbH, Germany). For this purpose, molds with 72 mm diameter and 22 mm height were first fabricated from condensation silicone impression material with putty consistency (Speedex, Coltene, Switzerland) and were filled with acrylic resin. The acrylic resin was prepared by mixing the powder and liquid as instructed by the manufacturer. Dental implants were mounted in a vertical position with a 90° angle relative to the acrylic surface using a surveyor (J. M. Ney Co., Bloomfield, CT, USA) and were allowed 24 h for the complete setting of the acrylic resin. Next, the abutments were cleaned in an ultrasonic bath at 40°C for 10 min and cleaned with water vapor for 4 min.

**Figure 1:**
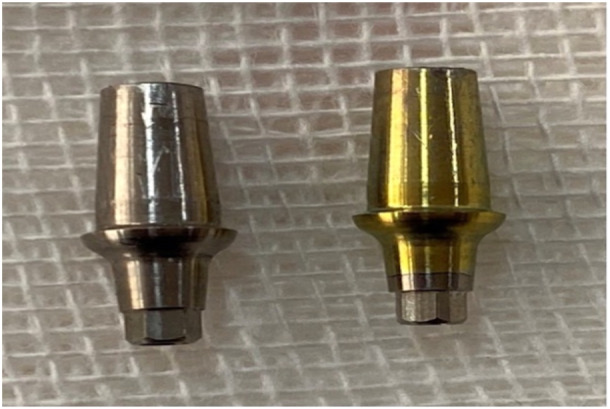
Anodized (right) and non‐anodized (left) abutments.

In the anodization group, nine abutments were anodized as follows: seven 9 V batteries (VDC digital bench power supply CS112001X Circuit Specialists, China) were connected in series to generate 63 V. A glass container was filled with 250 mL of distilled water and 1 g trisodium phosphate was added to it and allowed to dissolve. An electrolyte solution was prepared as such. The cathode was connected to a piece of aluminum foil measuring 6 × 3 cm and immersed in the electrolyte. The anode (red wire) was attached to the titanium abutment. During the anodization process, only the abutment was immersed in the solution, and care was taken to prevent the contact of the anode with the solution. The current entered the solution through the abutment. Next, the voltage was established, and anodization was performed for 5 s. This process was self‐limiting, and at 63 V voltage, a gold‐yellow color formed (Figure 1). Next, the anode was disconnected, and the abutments were inspected under adequate lighting and rinsed with acetone and deionized water.

**Figure 2:**
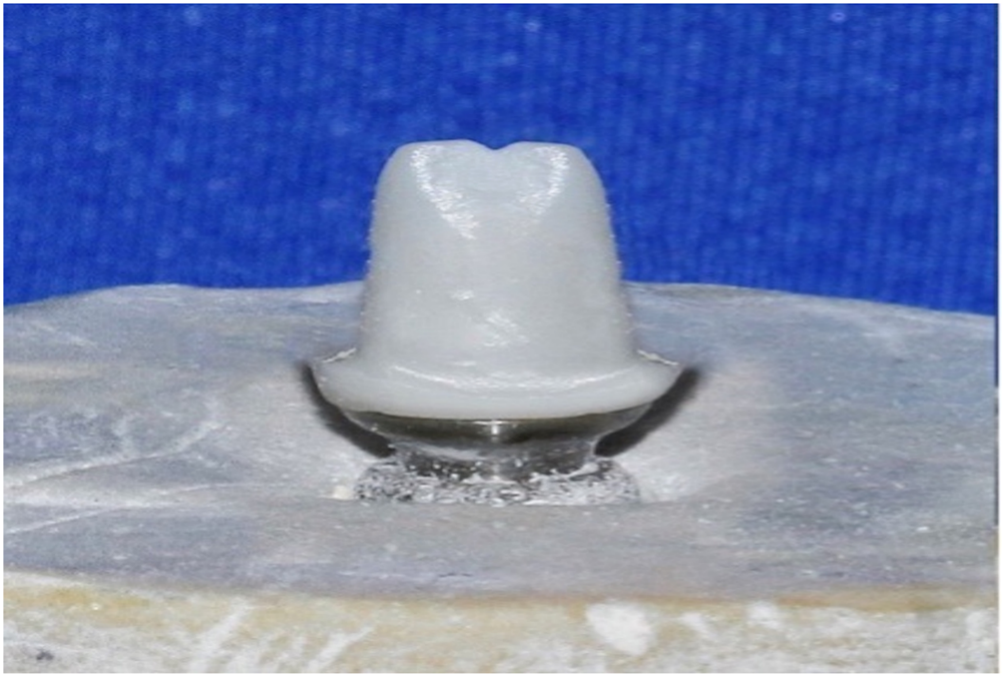
Zirconia hybrid abutment.

**Figure 3:**
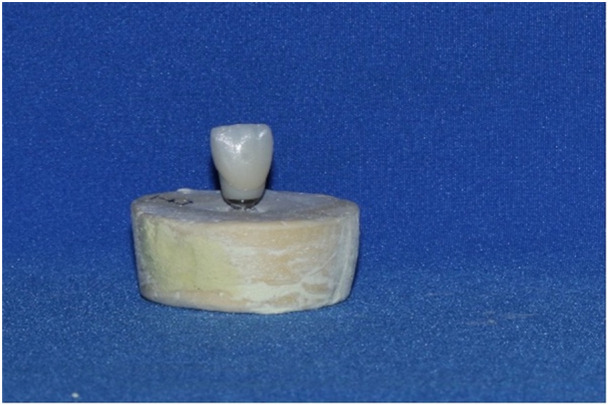
A mounted sample with LDS crown.

To fabricate the hybrid zirconia abutment as the standard sample (Figure 2), a titanium abutment was scanned by a scanner (Shining3D DS‐MIX, Shining 3D, China), and then an A2 shade zirconia block (Ceramil Zolid, Amann Girbach, China) was milled by a milling machine (Ceramil Motion2, Amann Girbach, China). Zirconia was initially in the monolithic phase. Thus, its volume was 20% higher than the volume of its final form. The sintering process was performed in a furnace (InFire HTC speed, Dentsply Sirona, USA) as instructed by the manufacturer. For the attachment of the zirconia structure to the abutment, surface treatment was performed with 50 µm aluminum oxide, followed by cleaning in an ultrasonic bath for 5 min and drying. One layer of metal primer (Alloy Primer, Kuraray Noritake Dental Inc., Tokyo, Japan) was then applied to the titanium surface. One layer of ceramic primer (Clearfil Ceramic Primer, Kuraray Noritake Dental Inc., Tokyo, Japan) was applied to the zirconia surface. Zirconia was attached to titanium using dual‐cure resin cement (Panavia F2.0, Kuraray Noritake Dental Inc., Tokyo, Japan), which was cured for 20 s. Next, the abutments were tightened on dental implants, and the abutment screw was torqued to 25 N/cm as instructed by the manufacturer using a torque meter (Tixos, Leader Italia, Italy). The specimens were then coded, and the abutments were scanned (Shining3D DS‐MIX, Shining 3D, China) for the fabrication of ceramic crowns. The resin pattern (Freeprint, DETAX, Germany) of a maxillary central incisor full‐contour monolithic crown with 2 mm thickness was designed using a software program (InLab SW18, Dentsply Sirona, USA) and printed by a 3D printer (Asiga 3D printer MAX, Asiga, Germany). The printed patterns were then sprued and placed in investment rings, and the cylinders were filled with investment gypsum (IPS Press Vest Speed Investment, Ivoclar, Schaan, Liechtenstein). After 60 min, the specimens were placed at room temperature to allow completion of the gypsum setting. The ring was then placed in the wax burn‐out furnace upside down as instructed by the manufacturer, heated to 850°C, and kept at this temperature for 60 min. After completion of preheating, the cylinder was placed in a heat‐press furnace (Programat P310, Ivoclar Vivadent, Liechtenstein) to prevent cooling. A cold A2 shade ceramic ingot (IPS E.max press; High Translucency, Ivoclar Vivadent, Liechtenstein) was placed on the opening of the warm investment facing up and subjected to load application by a cold IPS ALOX plunger. The furnace temperature increased from 700°C to 920°C within 22 min. The ceramic injection process took 4–5 min, and the heat pressing process of the ceramic was accomplished as such. After cooling to room temperature, the ceramic was removed from the investment. Excess material was carefully removed using diamond burs (Noritake Co., Japan), and the specimens were immersed in Invex liquid containing < 1% hydrofluoric acid for 10 min to eliminate the reaction layer. Finally, they were sandblasted with 50 µm aluminum oxide particles (Korox 50, Bego, Germany). The sprue was then cut with a diamond disc. For glazing, a 1:1 combination of IPS e.max Ceram glaze paste and IPS e.max Ceram glaze spray was used as instructed by the manufacturer. The specimens were then glazed in a furnace (Koushafan Pars, KFP, Iran) at 750°C for 1 min at a speed of 60°C/min and an initial temperature of 403°C. After fabrication, the crowns were cleaned in an ultrasonic bath (Woodpecker DTE D3, China) containing 96% isopropyl alcohol for 5 min. They were then placed over the abutments (Figure 3), and to ensure favorable optical contact, one drop of distilled water was applied between the ceramic and the abutment. For color measurement, the specimens were evaluated in three groups of standards (hybrid zirconia abutment + A2 shade LDS crown), control (non‐anodized abutment + A2 shade LDS crown), and test (anodized abutment + A2 shade LDS crown).

A spectroradiometer (CS‐2000; Konica Minolta Sensing Inc., Japan) was used to measure the color parameters with a 0.2° angle and 80 cm distance from the specimen to measure a circle with a 2.8 mm diameter at the center of each specimen. Two light sources illuminated the specimen surface at a 45° angle. A white tile was used for calibration (16176001, Calibration Plate, Japan). The specimens' L*, a*, b*, C*, and *h*° parameters were measured in the three groups. Each parameter was measured three times, and the mean of each parameter was calculated and recorded. L* (0 = black, 100 = white) indicated lightness/darkness, a* (−90 to 70) indicated redness (a* positive)‐greenness (a* negative), b* (−80 to 100) indicated yellowness (b* positive)‐blueness (b* negative), C* indicated chroma and *h*° indicated hue angle. To measure the color difference (Δ*E*_00_), the difference in the measured values in the test and control groups compared with the standard group was calculated. The CIEDE2000 (Δ*E*_00_) formula was used as follows:

$$\Delta E_{0}=\sqrt{\left(\Delta L\prime /KLSL\;\right)2+\left(\Delta C\prime /KCSC\;\right)2+\left(\Delta H\prime /KHSH\;\right)2+Rt\;\cdot \left(\Delta C\prime /KCSC\;\right)2\cdot \left(\Delta H\prime /KH\;SH\;\right)2.}$$

*T*‐test was applied to compare the color parameters of the groups using SPSS version 25 (SPSS Inc., Chicago, IL, USA) at 0.05 level of significance.

## 3 Results

Table 1 presents the measures of central dispersion of the color coordinates of the standard group (hybrid abutment along with LDS crown). Table 2 shows the measures of central dispersion of the color parameters of LDS crowns in anodized and non‐anodized groups.

**Table 1:** Measures of central dispersion of the color parameters of the standard group (hybrid abutment along with LDS crown).

Color parameter	Mean	SD	Minimum	Maximum
*L*	74.59	0.42	74.10	74.87
*a*	−0.49	0.09	0.58	0.39
*b*	17.14	0.15	17.01	17.30
*c*	17.14	0.14	17.02	17.30
*h*	91.62	0.33	91.28	91.94

**Table 2:** Measures of central dispersion of the color parameters of LDS crowns in anodized and non‐anodized groups.

Color parameter	Group	Mean	SD	SE	95% CI		Minimum	Maximum
					Lower bound	Upper bound		
*L*	Non‐anodized	73.22	2.45	0.47	72.25	74.19	68.65	76.59
	Anodized	74.95	0.77	0.15	74.65	75.26	73.25	76.26
*a*	Non‐anodized	1.08	0.27	0.05	0.97	1.19	0.52	1.37
	Anodized	1.36	0.19	0.04	1.29	1.43	1.03	1.69
*b*	Non‐anodized	16.42	1.45	0.28	15.85	16.99	13.69	18.53
	Anodized	17.44	0.52	0.1	17.23	17.64	16.68	18.72
*c*	Non‐anodized	16.38	1.47	0.28	15.79	16.96	13.70	18.58
	Anodized	17.51	0.53	0.10	17.29	17.72	16.72	18.77
*h*	Non‐anodized	86.36	0.75	0.14	86.07	86.66	85.23	87.81
	Anodized	85.54	0.59	0.11	85.31	85.78	84.60	86.58

Table 3 presents the measures of central dispersion for the change in color parameters of the anodized and non‐anodized groups compared with the standard group. According to the results of the regression test, significant differences were found in L* (*p* = 0.001), a* (*p* < 0.001), b* (*p* = 0.001), C* (*p* < 0.001), and *h*° (*p* < 0.001) parameters. The ΔL*, Δa*, Δb*, and ΔC* parameters in the anodized group were significantly higher than the corresponding values in the non‐anodized group. The Δ*h*° of the anodized group was significantly lower than that of the non‐anodized group.

**Table 3:** Measures of central dispersion for the change in color parameters of the anodized and non‐anodized groups compared with the standard group.

Color parameter	Group	Mean	SD	SE	95% CI		Minimum	Maximum	*p* value
					Lower bound	Upper bound			
*L*	Non‐anodized	−1.37	2.45	0.47	−2.34	−0.39	−5.94	2.0	*p* = 0.001
	Anodized	0.37	0.77	0.15	0.06	0.67	−1.34	1.67	
*a*	Non‐anodized	1.56	0.27	0.05	1.46	1.67	1.01	1.86	*p* < 0.001
	Anodized	1.85	0.19	0.04	1.77	1.92	1.52	2.18	
*b*	Non‐anodized	−0.71	1.45	0.28	−1.29	−0.14	−3.45	1.39	*p* = 0.001
	Anodized	0.29	0.52	0.1	0.09	0.51	−0.46	1.58	
*c*	Non‐anodized	−0.76	1.47	0.28	−1.35	−0.18	−3.44	1.44	*p* < 0.001
	Anodized	0.36	0.53	0.1	0.16	0.57	−0.42	1.63	
*h*	Non‐anodized	69.22	0.75	0.14	68.92	69.52	68.09	70.67	*p* < 0.001
	Anodized	68.39	0.59	0.11	68.16	68.63	67.46	69.44	

As shown in Table 4, the regression test revealed a significant difference in Δ*E*_00_ of the anodized and non‐anodized groups (*p* = 0.043).

**Table 4:** Measures of central dispersion for Δ*E*_00_ of all‐ceramic LDS crowns in anodized and non‐anodized abutments compared with the standard group.

Parameter	Group	Mean	SD	SE	95% CI		Minimum	Maximum	*p* value	Perceptibility threshold	Acceptability threshold
					Lower bound	Upper bound					
${∆E}_{00}$	Non‐anodized	2.98	0.51	0.17	2.58	3.36	2.4	3.89	0.043	0.8	1.8
	Anodized	2.26	0.2	0.06	2.11	2.41	1.94	2.55			

## 4 Discussion

In terms of color parameters, ΔL*, Δa*, Δb*, and ΔC* values in the anodized group were significantly higher than those in the non‐anodized group. However, Δ*h*° in the anodized group was significantly lower than that in the non‐anodized group. The present results showed significantly lower Δ*E*_00_ of LDS crowns placed over anodized abutments than Δ*E*_00_ of LDS crowns placed over non‐anodized abutments (compared with the standard sample) (Δ*E*_00_ = 2.26 vs. 2.98). This finding indicated the significant optimal effect of the anodization of titanium abutments on the color parameters of LDS crowns. Thus, the initial hypothesis of the study was confirmed.

The choice of a zirconia abutment as the standard sample in this study was based on its ability to allow deeper light emission and its minimal color difference compared to natural teeth, as supported by existing literature (Dede et al. 2016; Wang et al. 2019; Wadhwani et al. 2018; Peng et al. 2017; Vazouras et al. 2022; Martínez‐Rus et al. 2017). The choice of high‐translucency IPS e.max press was deliberate to mitigate the impact of ceramic translucency on masking the underlying titanium color. High‐translucency IPS e.max press is more sensitive to the color of the underlying tooth or abutment, enabling better color difference perception (Jirajariyavej, Wanapirom, and Anunmana 2018; Wang et al. 2019). However, it is essential to note that high translucency allows for greater light transmission and lower light reflection, increasing the risk of esthetic issues (Jirajariyavej, Wanapirom, and Anunmana 2018).

In the present study, the difference between the minimum and maximum values of all color parameters was smaller in the anodized group compared with the non‐anodized group, which indicates that the process of anodization was performed uniformly. The higher ΔL*, Δa*, Δb*, and ΔC* parameters in the anodized group indicated that the anodized samples were lighter, redder, and yellower than the non‐anodized group. Higher ΔC* indicates more color‐saturated specimens, while lower ΔC* indicates duller specimens. However, the lower values of Δ*h*° of the anodized suggest a shift to yellow in anodized specimens (Jirajariyavej, Wanapirom, and Anunmana 2018). Similarly, Farrag, Bakry, and Aly (2022a, 2022b) reported that yellow anodization of titanium increased the Δ*E*_00_, ΔL*, Δa*, and Δb* of high‐translucency LDS ceramic, compared with non‐anodized specimens.

The clinical acceptability and perceptibility threshold of Δ*E*_00_ for intraoral detection by the naked eye is 1.8 and 0.8, respectively. In the present study, Δ*E*_00_ values in both anodized (2.26) and non‐anodized (2.98) groups were clinically unacceptable; however, anodization decreased the color differences (although it was still clinically unacceptable). Vazouras et al. (2022) suggested pink anodized titanium abutments as a good alternative to hybrid zirconia abutments. Farrag and Khamis showed that anodized titanium abutments had no significant effect on the health or esthetics of peri‐implant soft tissue using the pink esthetic score (Farrag and Khamis 2023). Also, Wadhwani et al. (2018) indicated that pink anodized abutments had significantly lower color differences than non‐anodized abutments; however, they assessed the peri‐implant soft tissue esthetics and not LDS crowns. Several others reported the same results (Wang et al. 2019, 2021; Martínez‐Rus et al. 2017). In total, variations in the reported results can be attributed to variations in ceramic thickness and spectrophotometric variables (Dede et al. 2016, 2013).

Farrag, Bakry, and Aly (2022a) demonstrated that yellow anodization of titanium and increasing the ceramic thickness decreased color difference. They showed that a 1 mm thickness of high‐translucency LDS cannot mask the color of underlying titanium, even in the case of anodization of abutments. However, 1.5 mm ceramic thickness yielded clinically acceptable results only when applied over anodized abutments (1.7). However, the 2 mm thickness of LDS ceramic yielded acceptable results for both anodized and non‐anodized abutments (1.38 and 1.49, respectively). The same results were reported in another study by Farrag, Bakry, and Aly (2022b). The results of the abovementioned two studies were different from the present findings, which may be because they cemented the specimens with resin cement and used disc‐shaped specimens. Resin cement could affect the color of the crown (Elkhishen et al. 2022). Besides, temporary luting cements are the predominant choice for retaining implant prostheses (Mehta et al. 2023). They also used the Easy Shade spectrophotometer, which has an inferior performance compared to the device used in the present study. Jirajariyavej, Wanapirom, and Anunmana (2018) showed clinically unacceptable results in high‐translucency ceramics with < 2.5 mm thickness; however, a 2.5 mm thickness successfully masked the underlying color.

The present study used tooth‐shaped specimens instead of disc‐shaped specimens to simulate the clinical setting better. This was an advantage because the natural curvature of teeth can affect light absorption/transmission and, subsequently, the color parameters (Hung 1990).

This study offers valuable insights into the impact of anodization on titanium abutments in dental prosthetics, particularly regarding color matching. Demonstrating the effectiveness of anodization in reducing color differences provides practical guidance for clinicians aiming to improve esthetic outcomes in dental restorations. Additionally, the use of tooth‐shaped specimens enhances the study's clinical relevance. However, using one type of ceramic (LDS), one kind of translucency (high), and specific measurement points in the crown was a limitation of this study. Future studies are required on different ceramic types with different translucencies and different underlying substrate colors at different segments of the crown. The efficacy of cement in masking the substrate color was not evaluated in the present study either, which was another limitation. Also, due to the in vitro design of the study, the generalization of results to the clinical setting must be done with caution. Future studies are required on the effects of different cement colors, thicknesses, and translucencies on color match.

## 5 Conclusion

Within the limitations of this current study, it was concluded that:

- ΔL*, Δa*, Δb*, and ΔC* values were significantly higher in the anodized group compared to the non‐anodized group, indicating lighter, redder, yellower, and more color‐saturated anodized samples compared to non‐anodized ones.
- Δ*h*° in the anodized group was significantly lower than that in the non‐anodized group, with a shift to yellow in anodized samples.
- Δ*E*_00_ of LDS crowns placed over anodized abutments was significantly lower than that of LDS crowns placed over non‐anodized abutments. The findings suggest an optimal effect of the anodization of titanium abutments on the color parameters of LDS crowns.

## Ethics Statement

The School of Dentistry, Islamic Azad University, Tehran's ethics committee approved the study.

## Consent

The authors have nothing to report.

## Conflicts of Interest

The authors declare no conflicts of interest.

## Data Availability

The data that support the findings of this study are available from the corresponding author upon reasonable request.
